# Future Eldercare Planning Among Chinese Aging Families in Hong Kong

**DOI:** 10.1093/geroni/igab046.2028

**Published:** 2021-12-17

**Authors:** Xue Bai, Joanne Luk, Ranran He, Yanyee Kwong

**Affiliations:** The Hong Kong Polytechnic University, Hong Kong, Not Applicable, Hong Kong

## Abstract

Increasing attention has been paid to the potential role of care planning in buffering future eldercare challenges. However, little is known about the characteristics of care planning among Chinese ageing families. It is also of interest to reflect how recent events such as COVID-19 pandemic may affect their views of the future care planning. From a family systems perspective, this study explored the extent, processes, and contents of intergenerational care planning of Chinese ageing families in Hong Kong. Dyadic interviews were conducted with 60 adult child-older parent pairs, and individual interviews were conducted with another 33 adult children. Intergenerational discrepancies in extent and processes of care planning, intergenerational congruence of care expectations and struggles, facilitating role of family capital and hindering role of cultural capital in care planning were primary themes. Although both generations demonstrated strong awareness of future eldercare needs, they were found engaged in different levels and processes of care planning. Adult children’s level of engagement in planning activities may influence parents’ extent and contents of care planning. Intergenerational transmission of eldercare values contributed to intergenerational congruence of care expectations but also led to similar struggles and ambivalent attitudes toward future care. Moreover, family capital was found to facilitate family care planning while Chinese cultural values that emphasize family care may hinder both generations’ efforts in care planning. The findings will deepen our understanding on characteristics of intergenerational care planning in Asian Chinese communities and inform services to improve adult children and ageing parents’ preparation for future eldercare.

